# Practice nurse involvement in primary care depression management: an observational cost-effectiveness analysis

**DOI:** 10.1186/1471-2296-15-10

**Published:** 2014-01-14

**Authors:** Jodi Gray, Hossein Haji Ali Afzali, Justin Beilby, Christine Holton, David Banham, Jonathan Karnon

**Affiliations:** 1 Discipline of Public Health, The University of Adelaide, Adelaide, South Australia; 2 Faculty of Health Sciences, The University of Adelaide, Adelaide, South Australia; 3 Discipline of General Practice, The University of Adelaide, Adelaide, South Australia; 4 Office for Research Development, Health System Performance Division, SA Health, Adelaide, South Australia

**Keywords:** Depression, Practice nurse, Primary care, Collaborative care, Cost-effectiveness, RAC-E analysis

## Abstract

**Background:**

Most evidence on the effect of collaborative care for depression is derived in the selective environment of randomised controlled trials. In collaborative care, practice nurses may act as case managers. The Primary Care Services Improvement Project (PCSIP) aimed to assess the cost-effectiveness of alternative models of practice nurse involvement in a real world Australian setting. Previous analyses have demonstrated the value of high level practice nurse involvement in the management of diabetes and obesity. This paper reports on their value in the management of depression.

**Methods:**

General practices were assigned to a low or high model of care based on observed levels of practice nurse involvement in clinical-based activities for the management of depression (i.e. percentage of depression patients seen, percentage of consultation time spent on clinical-based activities). Linked, routinely collected data was used to determine patient level depression outcomes (proportion of depression-free days) and health service usage costs. Standardised depression assessment tools were not routinely used, therefore a classification framework to determine the patient’s depressive state was developed using proxy measures (e.g. symptoms, medications, referrals, hospitalisations and suicide attempts). Regression analyses of costs and depression outcomes were conducted, using propensity weighting to control for potential confounders.

**Results:**

Capacity to determine depressive state using the classification framework was dependent upon the level of detail provided in medical records. While antidepressant medication prescriptions were a strong indicator of depressive state, they could not be relied upon as the sole measure. Propensity score weighted analyses of total depression-related costs and depression outcomes, found that the high level model of care cost more (95% CI: -$314.76 to $584) and resulted in 5% less depression-free days (95% CI: -0.15 to 0.05), compared to the low level model. However, this result was highly uncertain, as shown by the confidence intervals.

**Conclusions:**

Classification of patients’ depressive state was feasible, but time consuming, using the classification framework proposed. Further validation of the framework is required. Unlike the analyses of diabetes and obesity management, no significant differences in the proportion of depression-free days or health service costs were found between the alternative levels of practice nurse involvement.

## Background

Globally, depression is the most common mental health disorder with a point prevalence between four and ten percent [1]. Depression has been identified as a health priority area in many countries, including Australia. It is estimated that 11.6% of Australians have experienced a depressive episode at some point in their life [2] and depression costs the Australian economy AU$14.9 billion per year [3]. Depression is the second most frequently managed chronic problem in Australian general practice [4].

Multiple systematic reviews and meta-analyses have found that collaborative care for depression is effective [5-7], and cost-effective [8,9]. Collaborative care involves a team based approach, where team members include the primary care physician (GP), a case manager (often a practice nurse) and a mental health specialist (e.g. psychiatrist, psychologist). The case manager’s role includes the systematic identification, management and follow-up of depressed patients. Adequate mental health training for the case manager was found to be a key determinant of effectiveness. The Australian Government offers financial incentives for general practices to employ practice nurses and to expand and enhance their role within the practice [10]. A practice nurse is a qualified nurse employed by a general practice to provide nursing management under the supervision of a general practitioner. The number of practice nurses in Australia increased from 3,255 in 2003–04 to 10,085 in 2009–10, with 58% of practices employing a practice nurse [11]. In 2009–10, Government practice nurse incentives totalled AU$55.3 million [12]. To date, studies of the Australian practice nurse workforce have been mainly descriptive, with little focus on models of practice or determining impact on health outcomes [13].

In Australia, the TrueBlue randomised control trial is the main study of practice nurse management of depression [14-16]. It examined clinical outcomes (e.g. reduction in depression score) associated with practice nurse led collaborative care for the management of moderate to severe depression comorbid with type 2 diabetes or coronary heart disease. The intervention comprised intensive training, supporting materials, access to a local facilitator, and monthly peer-support teleconferences. While both the intervention and control groups demonstrated a significant reduction in depression intensity after six months, the reduction was significantly larger in the intervention group.

As with the TrueBlue study, internationally, much of the evidence on the effectiveness of nurse involvement in collaborative care has been gathered via RCTs [5-7]. However, limitations exist, particularly when evaluating health care interventions with complex treatment pathways that are best represented as models of care. Questions remain about the external validity of RCTs conducted under highly controlled conditions with carefully selected patient populations [17]. Compared to RCTs, observational studies can offer greater external validity, and determine the effectiveness of interventions as applied in a real world setting.

Risk adjusted cost-effectiveness (RAC-E) is a method of analysis for identifying important differences in routinely provided services. RAC-E analysis has previously been applied to hospital services [18]. The Primary Care Services Improvement Project (PCSIP) was a retrospective observational study, which used RAC-E methods to assign general practices to models of care based on observed differences in practice nurse activity in the provision of clinical-based services. Three case studies were conducted [19,20], with this paper reporting on the depression case study.

RAC-E analysis uses routinely collected data to identify patient characteristics, and to track health outcomes and health care costs. Standardised assessment tools for depression are not consistently used within general practice or recorded in routine data. While previous studies have used proxy measures, such as antidepressant prescriptions, there is no clearly defined methodology to determine depressive state in the absence of standardised assessment tools or diagnostic interview [21-23]. As an illustrative case study of a RAC-E analysis, the PCSIP aimed to evaluate whether, from the perspective of the health-care system, a high level model of practice nurse involvement in the management of depression in primary care was more cost-effective than a low level model of practice nurse involvement. A secondary aim was to develop and test the feasibility of a depression state classification framework for use with routinely collected, general practice data. This paper describes the methodology developed to classify the depression status of patients, and reports the within trial cost-effectiveness findings of the RAC-E analysis.

## Methods

### Practice recruitment and classification of model of care

The recruitment of general practices and patients has been described in detail elsewhere [24]. Practices were recruited from within the Adelaide Northern Division of General Practice (ANDGP), which is located in the northern suburbs of metropolitan Adelaide, South Australia. All 66 practices in the ANDGP were contacted. Ten practices with practice nurses agreed to participate in the PCSIP.

Practice nurses within the participating practices were surveyed to determine their level of involvement in clinical-based activities in the management of depression (e.g. patient education, self-management advice, monitoring of treatment adherence). Level of involvement was determined based on two questions within the survey: (1) What is your best estimate of the percentage of patients with DEPRESSION that are seen by you [i.e. the practice nurse] in your practice? (2) In an average consultation with a DEPRESSED patient, what percentage of time do you spend providing education and self-management advice, monitoring clinical progress, and assessing and enhancing treatment adherence? Where the average response of the practice nurses within a practice was greater than 50% on both these questions, the practice was considered to show a high level of practice nurse involvement and assigned to the high level model of care. If high level criteria were not met, the practice was assigned to the low level model of care.

To identify any further differences between the models of care, the following were also analysed: responses to other questions in the practice nurse survey regarding the practice environment and practice nurse characteristics (e.g. age, gender, education and experience); billing of Medicare service item number 10997, which covers ‘provision of monitoring and support for a person with a chronic disease by a practice nurse or Aboriginal and Torres Strait Islander health practitioner’ [25]; and the billing of Medicare mental health service item numbers (2702, 2710 and 2712), which covered preparation and review of mental health care plans by GPs.

### Patient recruitment

Within participating practices, eligible patients were identified using the Pen Computer Systems Clinical Audit Tool (CAT). The CAT tool identified patients who had a diagnosis of depression listed in their medical history summary. This meant we were more likely to select patients who had experienced depression severe enough to warrant a diagnosis and ongoing monitoring or intervention, rather than patients with a milder, transient experience of ‘feeling depressed’ (which would be recorded in the general consultation notes). Eligible patients were aged between 18 and 75 years, regularly visited the practice (i.e. at least 3 times in the last 2 years), were not under regular psychiatric care, not pregnant, not living in a managed care facility and did not have a severe mental disorder or mental impairment (e.g. schizophrenia, bipolar disorder, or dementia). After recruitment, but prior to data analysis, eligibility criteria were further refined to exclude patients for whom less than 50% of their GP visits during the study period were to the participating practice (as indicated by Medicare billing of GP visit item numbers). Patients were asked for consent to access their medical records held by the participating general practice, Medicare Australia (the federal government department which organises and distributes payment for Australia’s publically funded, universal health care system) and SA Health. There was no intervention within the study design. Sample size calculations estimated that 100 patients per model of care were required, details of calculations have been provided elsewhere [24].

Ethics approval was granted by the Human Research Ethics Committees of the University of Adelaide and SA Health (the South Australian Department of Health).

### Data sources and data collection

Data were collected for the period between October 2007 and October 2010 from three sources: patient medical records held at general practices, Medicare Australia, and SA Health.

Patient medical records provided information on patient characteristics (e.g. age, gender, comorbidities), referrals to specialists and allied health professionals, prescriptions written, GP management plans prepared or reviewed, scores on any standardised assessment tools for depression and general medical notes. Record information was extracted from the practice and entered directly into a purpose built Access 2007 database (Microsoft Office 2007, Microsoft Corporation), with identifying information removed.

Medicare Australia provided data on out-of-hospital health service usage and costs, including GP visits, management plan preparation or review, psychological services provided under the Better Access Initiative, specialist visits, and prescriptions provided under the government subsidised Pharmaceutical Benefits Scheme (PBS). Unit costs were allocated as per the year incurred. SA Health provided information on public and private inpatient hospital services, to which 2008–09 average diagnosis related group (DRG) costs were applied. The evaluation took the perspective of the health-care system, thus only direct health-care costs were included.

Data from each source were cleaned, formatted and linked to create comprehensive individual patient records.

### Classification of depression state

To track the progression of depression, the depression state of each patient needed to be categorised throughout the study period. Standardised assessment tools for depression, such as the DASS-21 or the Hamilton Rating Scale for Depression, were not routinely used or recorded within the obtained data, therefore proxy measures had to be developed.

**Figure 1:**
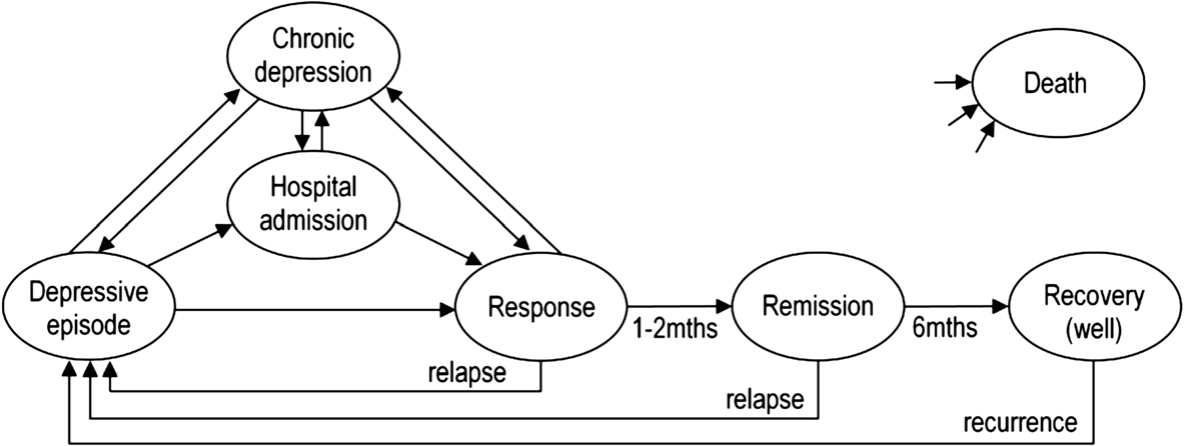
**Depression model showing depressive states and transitions.** Ovals indicate states, arrows indicate transitions between states. Patients may transition into the death state from any other state (i.e. all cause mortality). Adapted from [23].

A proposed model structure (Figure 1) for cost-effectiveness analyses of depression management includes six clinically and economically relevant depression-related states, based on the natural history of depression [23].

Howell [26] published a ‘Case note audit form’, which was used to identify relapse in depression patients from general practice medical records. The audit form contained detailed lists of depression symptoms, treatment options, depression-related medications and mental health services to which a patient could be referred. In a prior publication [23], we suggested a method of incorporating changes to treatment and GP notes as proxy measures from which to determine a range of depressive states beyond relapse. In practice the proposed method required simplification in order to apply, resulting in the following conceptualisation of the symptoms and timing of depressive states:

• Depressive episode – significant symptom intensity described, with impact on daily living.

• Chronic depression – low or moderate symptoms of depression are recorded and appear to have been present for two or more years.

• Hospital admission – where the reason for admission is depression-related. Reason for admission may be determined via general notes in the GP records, discharge summaries provided to the GP, or diagnosis related data provided by SA Health.

• Response – patient experiences a substantial reduction in depressive symptoms (e.g. symptoms appear to ‘halve’). Response period is considered to last a minimum of eight weeks, but may last longer if symptoms remain reduced but do not fully abate.

• Remission – patient is symptom free or experiences very minimal symptoms. Remission is considered to last for six months following response.

• Recovery – a patient who remains asymptomatic and has spent six months in the remission state is considered to transition into the recovery state.

In addition to the symptom intensity and timing described above, antidepressant medications, psychology and psychiatry referrals, emergency department referrals or suicide attempts were also incorporated. Further details on the classification process are provided in the appendix (see Additional file 1).

While the analyses described in this paper ultimately required the classification of only two states – depression or depression-free – it was intended that the dataset would also be used to populate a decision analytic model based on the proposed model structure. Hence, it was important that the classification system be able to identify all six depressive states shown in Figure 1, and that the proposed model structure was congruent with the observed data.

### Analysis

All analyses were undertaken using STATA, Release 12 (StataCorp). The duration of a patient’s participation in the study was calculated as the number of days between the first and last visits at which the patient’s depressive state could be determined.

The number of depression-free days were calculated as the total number of days spent in either the remission or recovery states. In all other states, including response, the patient experienced some degree of depression. For each patient, the proportion of depression-free days (pDFDs) were calculated as the total number of depression-free days, divided by the number of days the patient participated in the study. Health service costs were calculated per patient for the participation period.

Both unadjusted and adjusted multiple regression analyses were undertaken to determine differences between models of care in the pDFDs experienced and health service costs. To adjust for potential confounders, propensity score weighted analyses were used [27]. Variables considered as potential covariates in the logistic regression model included: patient age, gender, marital status, socioeconomic status (based on postcode level census data (SEIFA score)), concessional status, relevant physical comorbidites (myocardial infarction, ischemic heart disease, congestive heart failure, stroke, cancer, diabetes, respiratory disease, musculoskeletal conditions and chronic pain), relevant psychological comorbidities (alcohol or drug abuse, gambling addiction, postnatal depression, anxiety disorder, post traumatic stress disorder, obsessive compulsive disorder, eating disorders, and social or other phobia), measures of practice loyalty (the length of time since the patient first attended the practice, the proportion of visits to a GP during the study that were at the participating practice, and the number of practices attended during the study), depression history (the length of time since the first recorded depressive episode (which may or may not have been prior to the study period), and the depressive state at the start of the study period) and the number of days the patient participated in the study.

The distribution of pDFD contained distinct peaks at both 0 and 1, and so a propensity weighted zero one inflated beta (ZOIB) model, with clustering of patients by practice, was used for the adjusted analysis [28,29]. This model contains three components: two separate logistic regression models to predict whether the proportion is equal to 0 or 1, and a beta model to predict proportions between 0 and 1. Variables considered as potential covariates included those listed above, as well as GP characteristics (age, gender, experience, and the number of training sessions in depression undertaken in the previous two years), practice characteristics (bulk billing behaviour, practice size as measured by the number of GPs, and the number of depression patients attending the practice (defined as the number of eligible depression patients identified during the patient recruitment CAT search)) and the model of care. Model fit was determined using the Ramsey RESET test.

Adjusted analyses of health service costs were undertaken, by fitting generalised linear models (GLMs), which allowed for weighting using average treatment effect (ATE) weights [27] and clustering by general practice. The same variables, as listed for the ZOIB model, were considered as potential covariates. Goodness of fit was determined using the modified Park test (for the GLM family) and the Pearson correlation test, the Pregibon link test, and the modified Hosmer and Lemeshow test (for the GLM link) [30]. Total costs were analysed including and excluding hospital costs, to determine whether hospital costs were an important driver of expenditure. A bootstrapping approach (one thousand bootstrap samples) was applied to represent the uncertainty around the mean outcome and cost estimates.

## Results

Ten general practices with practice nurses were recruited to the PCSIP. One practice was later excluded as only one patient with depression could be recruited from this practice. On the basis of the practice nurse survey, six practices were allocated to the low level model of care and three to the high level model. Across the nine practices, 208 depression patients were initially recruited. The response rates were 33% (124 patients) and 30% (84 patients) for the low and high level models of care, respectively.

### Depression state classification

During data extraction and the subsequent classification process, 54 of the 208 recruited patients (25 from the high level model, 29 from the low level model) were excluded from the study. Exclusions occurred as it became apparent from the extracted data that these patients did not meet the defined inclusion criteria (e.g. they were not depression patients but instead had a primary diagnosis of anxiety or had experienced symptoms indicative of psychosis, received regular psychiatric care, were living in managed accommodation, the participating practice was not the patients’ main practice, or the first categorised visit was beyond the study period).

The level of detail provided in medical notes regarding the patients’ experience of depression symptoms and their severity varied between practices and GPs. Chronic depression was particularly difficult to identify, while depressive episodes, having higher symptom intensity and greater functional impairment, appeared to be better documented. In instances where medical notes were less detailed, some states had to be inferred. An assumption was made that symptoms were not present if not recorded, however the reality may be that symptoms remained at lower levels. Where patients stated they had experienced ‘long standing depression’, details of the depression history were generally not provided, and we were unable to determine if the patient experienced episodes of depression interspersed with periods of wellness, or prolonged periods of depression with unremitting symptoms (i.e. chronic depression).

Where present, distressing life events (both current and historical) and comorbid conditions (such as anxiety, alcoholism, chronic pain or musculoskeletal injury) often appeared to interact with, exacerbate or share symptoms with depression. Thus it was challenging to differentiate the course of a comorbidity from the course of a depressive episode, particularly if, after initially noting concurrent depression, the medical notes focussed predominantly on the comorbidity.

While changes to antidepressant prescriptions provided a strong indicator of the course of a depressive episode, limitations applied. Despite being offered, some patients experiencing a depressive episode were unwilling to take antidepressant medications. Some antidepressants and mood stabilising medications were prescribed for non-depression-related reasons, for example amitriptyline for management of neuropathic pain or sodium valproate for epilepsy. Patient compliance varied, with some patients ceasing, decreasing or increasing dosages independent of medical advice. Medications could be ceased or dosages reduced due to side-effects or affordability, rather than symptom abatement, and reasons for changes were not always provided. These findings suggest that antidepressant prescriptions have limited use as a sole indicator of depressive state.

While most patients followed pathways congruent with the proposed depression model structure (Figure 1), three did not. One patient experienced a rapid onset of depression and anxiety symptoms due to life events, resulting in a transition directly from the recovery (well) state to a hospital admission. Two patients transitioned from a hospital admission to a depressive episode, rather than the response state as proposed. No patients died during the study period.

### Practice, GP and practice nurse characteristics

Characteristics of practices and practice nurses are shown in Table 1. While no practice nurses reported specific qualifications in the field of mental health care, practice nurses in high level model practices attended significantly more training sessions for depression in the previous two years (1.33 sessions), compared with practice nurses in low level model practices (0.38 sessions, p = 0.04). No other significant differences in practice or practice nurse characteristics were found.

**Table 1:** Practice, GP and practice nurse characteristics by model of care

	**Low level model**	**High level model**	**p value**
**Practice characteristics (mean)**			
Number of practices in model	6	3	
Number of GPs	6.00	2.67	0.11
Total number of patients with depression	398.83	235.00	0.47
GP age (years)	45.70	46.11	0.94
GP experience (years)	19.04	20.89	0.77
GP gender (proportion male)	0.70	0.72	0.93
Number of depression-related training sessions attended by GPs in last 2 years	1.69	2.11	0.42
Practices bulk bill^1^ (proportion):			
all patients	0.50	1.00	
concession/pension card patients only	0.50	0.00	0.13
Billing of Medicare mental health item numbers (items per patient year)^2^	0.37	0.34	0.78
**Practice nurse (PN) characteristics (mean per practice)**			
Number of PNs in model	13	3	
Number FTE^3^ PNs per GP	0.24	0.27	0.70
PN age (years)	49.92	46.33	0.40
Experience working as a PN (years)	6.20	9.17	0.55
Experience working as a PN in the participating practice (years)	5.13	3.50	0.68
Number of depression-related training sessions attended by PNs in last 2 years	0.38	1.33	0.04
Billing of Medicare item number 10997 (items per patient year)^4^	0.08	0.06	0.64

^1^The Medicare benefit paid for the visit is accepted as the full fee, therefore there is no out of pocket expense for the patient. ^2^Included mental health item numbers cover preparation of a GP mental health care plan by a medical practitioner (2702, 2710), and review of a GP Mental Health Treatment plan (2712). ^3^FTE: full time equivalent. ^4^Item number 10997 covers ‘provision of monitoring and support to people with a chronic disease by a practice nurse or registered Aboriginal Health Worker on behalf of a GP’.

### Patient characteristics

Patient characteristics for the 99 patients from low level model practices and 55 patients from high level model practices participating in the PCSIP are shown in Table 2. In the unweighted (before adjustment) analysis, significant differences (p < 0.05) existed between the models in terms of the socioeconomic status of the patient, the duration of attendance at the participating practice, and duration of study follow-up.

**Table 2:** Patient characteristics, before and after propensity score weighting

	**Before adjustment**			**After adjustment**^**1**^		
	**Low level model**^**2**^	**High level model**^**2**^	**St. difference (p value)**^**3**^	**Low level model**	**High level model**	**St. difference (p value)**
Age (years)	52.56	51.14	0.12 (0.49)	50.13	51.00	-0.07 (0.70)
Gender (male)	0.24	0.28	-0.11 (0.53)	0.19	0.25	-0.14 (0.37)
Married or defacto relationship	0.49	0.38	0.22 (0.20)	0.40	0.40	-0.001 (0.996)
Concessional patient	0.80	0.68	0.28 (0.10)	0.66	0.70	-0.10 (0.68)
SEIFA score^4^	854.24	905.08	-0.70 (0.00)	877.86	887.05	-0.13 (0.60)
Time since first recorded depressive episode (days)	824.91	1226.00	-0.32 (0.06)	1068.46	1135.63	-0.05 (0.78)
In a depressive state at start of study^5^	0.60	0.52	0.17 (0.31)	0.66	0.56	0.20 (0.26)
Pre-study chronic condition^6^	0.58	0.52	0.13 (0.43)	0.51	0.51	-0.01 (0.96)
Pre-study psychological condtion^7^	0.15	0.18	-0.10 (0.57)	0.16	0.17	-0.03 (0.89)
Time attending the practice (days)	816.58	1564.69	-0.65 (0.00)	1118.51	1280.96	-0.14 (0.52)
Percentage of GP visits in the study period to the participating practice	0.94	0.93	0.15 (0.39)	0.94	0.93	0.11 (0.50)
Time in study (days)	799.02	882.05	-0.38 (0.02)	812.96	843.78	-0.14 (0.54)

^1^Adjustment is based on propensity score weighted analysis. ^2^Low level model includes 99 patients, high level model includes 55 patients. ^3^Standardised difference is the difference between the means for the low and high level practices divided by the standard deviation (p value is for differences). ^4^SEIFA is the Socio-Economic Index for Areas (lower scores indicate more disadvantage, Australia wide scores are standardised to a mean of 1000). ^5^Depressive states include depressive episode, chronic depression, hospital admission for depression, or response. ^6^Pre-study chronic conditions include myocardial infarction, ischemic heart disease, congestive heart failure, stroke, cancer (excluding skin cancer), diabetes, respiratory disease (e.g. asthma, COPD), musculoskeletal conditions (e.g. arthritis) and chronic pain. ^7^Pre-study psychological conditions include alcohol or drug abuse, gambling addiction, postnatal depression, anxiety disorder, post traumatic stress disorder, obsessive compulsive disorder, eating disorders, and social or other phobia.

The application of propensity weights reduced differences between the two models for all but two patient characteristics (gender, and whether the patient was in a depressive state at the start of the study) and attained standardised differences of less than 0.1, indicating negligible differences [31], for five of the twelve characteristics. The subsequent use of propensity weighted regression further controls for those variables with standardised differences greater than 0.1.

### Outcomes and costs

Unadjusted analyses found no significant differences in the pDFDs between the high and low level models of care (Table 3). Medicare out-of-hospital costs were significantly higher for the high level model compared to the low level model (high level = $2039, low level = $1502, p = 0.005), as were total depression-related costs (high level = $2374, low level = $1750, p = 0.01).

**Table 3:** Unadjusted and adjusted costs and outcomes per alternative models of care

	**Before adjustment**				**After adjustment**^**1**^	
**Variables**	**Low level model**	**High level model**	**High level minus low level model mean difference (95% CI)**	**p value**	**High level minus low level model mean difference (95% CI)**	**p value**
Proportion of depression-free days (mean)	0.55	0.51	-0.04 (-0.17 to 0.09)	0.54	-0.05 (-0.15 to 0.05)	0.31
Medicare out-of-hospital costs	$1502	$2039	$537 (165 to 909)	0.005	$33 (-270 to 337)	0.83
Total pharmaceutical^2^ costs	$1185	$1389	$204 (-340 to 749)	0.46	$147 (-161 to 456)	0.35
Total depression-related pharmaceutical costs^3^	$156	$171	$14 (-50 to 79)	0.65	$41 (-7 to 89)	0.09
Total hospital costs	$1964	$1525	-$439 (-1513 to 636)	0.42	$181 (-742 to 1103)	0.70
Total depression-related hospital costs	$92	$164	$72 (-206 to 351)	0.61	---	---
Total costs	$4652	$4954	$302 (-1155 to 1760)	0.68	$574 (-487 to 1635)	0.29
Total depression-related costs^4^	$1750	$2374	$624 (150 to 1098)	0.01	$135 (-315 to 584)	0.56

^1^Adjustment is based on propensity score weighted analysis. ^2^For all pharmaceuticals supplied under the Pharmaceutical Benefits Scheme (PBS). ^3^Antidepressant medications supplied under the PBS. ^4^Total depression-related costs = Medicare out-of-hospital costs + total depression-related pharmaceutical costs + total depression-related hospital costs.

**Figure 2:**
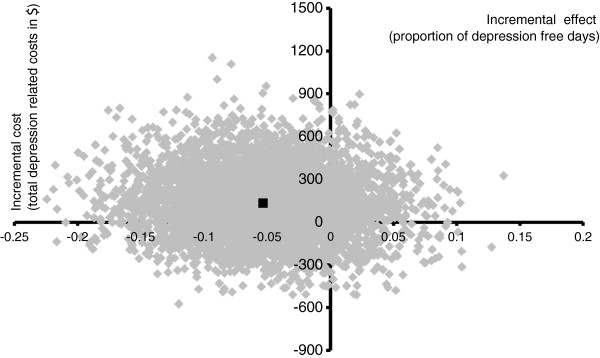
**Cost-effectiveness plane.** Incremental (high level minus low level model) costs and effects after adjustment. Black square shows mean values.

Adjusted analyses found no significant difference in pDFDs or costs between the models of care (Table 3). Based on the mean adjusted estimates of the primary cost measure (total depression-related costs) and outcome variable (proportion of depression-free days), the high-level practice nurse model of care was shown to cost more and resulted in 5% less depression-free days than the low level model. However, this result is highly uncertain. A bootstrapped sensitivity analysis generated a 95% confidence interval that ranged from 15% less to 5% more depression-free days for the high level model of care, with a cost difference ranging from minus $314.76 to $584.00 more. A cost-effectiveness plane, showing five thousand bootstrapped samples, was generated to demonstrate the uncertainty (Figure 2). While the sampled values spread across all four segments of the plane, 62% are positioned in the north west quadrant, in which the high level model is more costly and less effective than the low level model.

## Discussion

This study has reported on the application of a framework to classify depression-related states of health using routinely collected clinical data from a range of sources. The resulting classifications informed a cost-effectiveness analysis of alternative levels of involvement of practice nurses in the care of patients with depression in an Australian primary health care setting.

The process of manually categorising depressive state was time consuming, but feasible using the protocol described. Almost all observed transitions between states followed pathways consistent with the proposed depression model, suggesting strong congruence between the proposed model structure and the observed data.

Variation in the level of detail provided in general practice medical notes may have been a source of potential bias, especially as it was necessary to assume that the absence of notes regarding depressive symptoms indicated the absence of symptoms. This is of particular concern for chronic depression, where persistent low to moderate level symptoms may be less likely to be recorded, especially by GPs who are providing a lower intensity of depression care.

In future applications, the inclusion of a measure of uncertainty for each classified state may be one way of quantifying bias in the classification process. For example, adding a notation on whether a state was allocated based on multiple indicators, a single indicator or via time-based assumptions only.

The classification framework expands on methods used in previous studies, which have classified depressive state based solely on the antidepressant prescription information available in routinely collected medical records [21,22]. For example, Sicras-Mainer *et al.*[22] defined patients as entering remission after completing six months of antidepressant therapy. Discontinuation of the antidepressant was not considered evidence of remission as patients may discontinue for other reasons, or continue treatment for prolonged periods despite being asymptomatic. Comparing remission identified via antidepressant use and remission identified through a review of a random sample of patient medical records, Sicras-Mainer *et al.* found high concordance between the two measures. Details of how remission was determined in the medical record review were not supplied. Nordstrom *et al.*[21] determined depressive relapse on the basis of an antidepressant prescription received within one to six months of ceasing an antidepressant.

The PCSIP found that, while changes to antidepressant prescriptions provided an indication of depressive state, there were limitations to using them as a sole means of classification. For example, cross-referencing Medicare and general practice data found that not all prescriptions written were supplied under the PBS. Patient medical notes also indicated that not all antidepressant prescriptions were written to treat depressive symptoms. This is consistent with the findings of the Bettering the Evaluation and Care of Health (BEACH) programme [32], which continually surveys general practice activity across Australia. The BEACH programme found only 70% of antidepressant prescriptions were for depression. The remaining 30% were prescribed for other psychological issues, such as anxiety, phobias, or eating disorders, or for non-psychological issues, such as musculoskeletal and neurological problems.

Further work is required to validate the developed classification framework. This will require a comparison of classifications based on routinely collected data sources with classifications based on either diagnostic interview of patients or responses to standardised depression assessment tools taken independently of routine practice at regular time intervals. Threshold values on these tools are commonly used to define depression states (such as response, remission, recurrence) in RCT based modelling studies [33,34].

Strengths of the PCSIP include: the assessment of the level of practice nurse involvement, rather than assessing only the presence or absence of a practice nurse; the allocation of general practices to models of care based on existing differences, rather than through the imposition of an intervention, allowing the study to examine the real world impact; and the classification of depression state using the framework derived, rather than relying on medication changes alone.

The study was subject to some limitations, including: the relatively small sample size, particularly given the high level model did not reach the numbers indicated by the sample size calculation; the allocation of practices to models of care based on the subjective responses of practice nurses; and the observational study design which, despite the rigorous methods used to minimise bias due to observed confounders (i.e. combined use of propensity score weighting and regression analyses), still had an associated likelihood of unobserved confounding between the two models of care (for example, unknown differences in the number of previous depressive episodes, or occurrence of stressful life events). Unobserved differences in the characteristics of patients consenting and not consenting to participate in the study may have resulted in a selection bias, which would reduce the representativeness of the sample population to the general depression population. Cost values were not discounted over the three year time horizon and Medicare costs remained in the unit cost of the year incurred, rather than being re-estimated for a single base year. Given the relatively short time horizon of the study, the consistency in cost allocation methods across the models of care and the small difference in costs found, this is unlikely to have had a significant effect on the findings of the study.

In interpreting the finding of no significant difference in costs and outcomes between the models of care it is pertinent to consider that, although practice nurses in the high level model attended more depression related training sessions, none of the practice nurses in the study had formal qualifications in mental health care or experience working in a mental health nurse role. Information was not collected on the duration of training sessions attended, however, at the time of the study, the majority of training sessions for practice nurses were provided by the ANDGP. These consisted of evening education sessions lasting approximately two and a half hours (ANDGP, personal communication, 17 May 2011). Therefore, the actual difference in the number of hours of training may equate to as little as two and a half hours over the two years. Thus, the result may reflect similar, low levels of mental health training across both models of care. Although the study did not include a ‘no practice nurse’ model of care, the findings imply that practice nurses with low levels of training in mental health have a limited effect on the outcomes of patients with depression.

Existing evidence indicates that the inclusion of case managers, who are often nurses, in collaborative care for depression is effective [5-7] and cost-effective [8]. However, it is vitally important that nurses engaged in this role receive adequate training and ongoing support [5,7]. Effectiveness is further improved if the nurse is able to provide psychological therapies as a part of enhanced care [6]. For example, the IMPACT method includes 4 days of training, 30 sessions with ‘training patients’ and 15 supervised sessions with videotape review. The nurse is then able to provide patient education, discuss treatment options, conduct follow-up and deliver a 6 to 8 session psychotherapy based intervention [35]. The TrueBlue study, which translates the IMPACT methodology to the Australian setting, provides 2 days of training, though this covers chronic disease management for diabetes and heart disease, as well as depression care (screening and counselling). Practice nurses were also given case management templates that provided written protocols for “gold-standard” depression management, as well as ongoing expert and peer support [14-16]. Since 2007, the Mental Health Nurse Incentive Program (MHNIP) has provided incentives for Australian general practices, private psychiatry practices and Indigenous health services to employ a credentialed mental health nurse [36]. In 2012, there were 1,153 credentialed mental health nurses in Australia and the MHNIP had been taken up by 470 organisations [37]. Unfortunately, none were employed in the ANDGP area at the time of the PCSIP to enable comparison with generalist practice nurses.

Despite the limitations, and in line with previous studies, the PCSIP findings suggest that practice nurses involved in the management of patients with depression require specific training in mental health. They suggest that without focused training and support, it is not an efficient use of scarce nursing time to promote greater involvement of generalist nurses in the care of depressed patients. Training may range from two days (the TrueBlue study) to a university awarded postgraduate qualification with additional work experience (mental health nurse credential required to receive the MHNIP). Other RAC-E studies have shown that greater practice nurse involvement in the care of patients with diabetes and obesity is cost-effective [19,20]. Thus, in primary care settings where no specific framework for mental health training and support has been provided, practice nurse time might be better targeted towards diabetic or obese patient groups.

The general methods used in the PCSIP, including the application of the RAC-E methodology and the depression state classification framework, demonstrate the feasibility of evaluating real world interventions in the primary care setting. The non-significant differences found in outcomes and costs illustrate the value of such evaluations, particularly for those involved in policy and planning decisions, such as expansion or redirection of financial incentives.

## Conclusions

This study has shown that it is feasible to classify depression status based on routinely collected clinical data collated from multiple sources. Further research is required to validate the described classification process, and findings should be interpreted with some caution until this has been done.

This paper used data from a retrospective observational study based on routinely collected data. It has reported small and non-significant differences in health service costs and proportions of depression-free days between patients attending general practices in which practice nurses were defined as having a high level of involvement in the clinical care of depression patients, and practices with a low level of practice nurse involvement. Further research might focus on the costs and effects of different levels of training and support for practice nurses in a mental health role and the use of specialist mental health nurses in routine general practice.

## Abbreviations

AD: Antidepressant; ANDGP: Adelaide Northern Division of General Practice; ATE weights: Average treatment effect; DRG: Diagnosis related group; ED: Emergency department; FTE: Full time equivalent; GLM: Generalised linear model; GP: General practitioner; MHNIP: Mental Health Nurse Incentive Program; PBS: Pharmaceutical Benefits Scheme; PCSIP: Primary Care Services Improvement Project; pDFDs: Proportion of depression-free days; PN: Practice nurse; RAC-E: Risk adjusted cost-effectiveness; RCT: Randomised controlled trial; SA Health: The South Australian Department of Health; SEIFA: Socio-Economic Index for Areas; St. difference: Standardised difference; ZOIB: Zero one inflated beta.

## Competing interests

There are no known competing interests for any of the authors in relation to the work presented in this paper.

## Authors’ contributions

All authors contributed to study design, interpretation of results and drafting of manuscript. In addition: JK was responsible for study conception and organisation; JG performed data extraction; HH conducted the classification of model of care; HH and JG designed the classification framework and performed depression state classification; JG performed the statistical analysis, advised by JK and HH; JG completed the first draft of the manuscript. All authors read and approved the final manuscript.

## Pre-publication history

The pre-publication history for this paper can be accessed here:

http://www.biomedcentral.com/1471-2296/15/10/prepub

## Supplementary Material

Additional file 1 **Appendix: The process of classifying depressive state.** The appendix provides additional details on the process of classifying the depressive state of the patients, using the proposed framework. Click here for file
