# Supplementary material for: Practice nurse involvement in primary care depression management: an observational cost-effectiveness analysis
Source: BMC Fam Pract. 2014 Jan 14;15:10. doi: 10.1186/1471-2296-15-10 (PMC3897884; doi:10.1186/1471-2296-15-10)
Supplement: Additional file 1 — Appendix: The process of classifying depressive state. The appendix provides additional details on the process of classifying the depressive state of the patients, using the proposed framework. [file 1471-2296-15-10-S1.docx]

**Appendix: The process of classifying depressive state**

**Classification of depressive state**

An Access 2007 database (Microsoft Office 2007, developed by Microsoft Corporation) was developed specifically for the classification process. It enabled researchers to view the data for individual depression patients, sorted in chronological order. Where a GP visit contained information relevant to mental health status, the database displayed full details of the visit (i.e. notes, prescriptions, referrals, mental health care plans). Visible alongside this was overview data for the patient. Overview data was organised by date to create a timeline which included:

- dates of all GP visits, whether relevant to mental health or not
- dates of all visits to mental health professionals, as noted in medical records or billed through Medicare
- hospitalisations, as recorded in the medical notes
- all antidepressant or anti-anxiety prescriptions written by the GP, with dosages
- all antidepressant or anti-anxiety prescriptions supplied under the PBS
- scores on any standardised depression assessment tools used, and
- the diagnosis of any new comorbidities.

Researchers sequentially viewed the detailed GP visits and took into account additional information from the timeline, to determine if any of the events listed in Table 1 had occurred. Within the timeline, drop down menus allowed researchers to classify the depressive state of the patient at each time point, and enter a reason for the classification. Reasons were grouped by changes to medications, psychology or psychiatry referrals, and symptoms or other indicators. Additional time points could be added, for example where GP notes indicated onset of symptoms at a prior date. Classification for all patients was initially conducted by one researcher, who was provided education on depression prior to commencing. Classification decisions were then reviewed by a researcher who is also a qualified GP. Disparities in classification were discussed until a consensus was reached.

**Classification assumptions**

Due to limitations in the available data, particularly the lack of details on the patients depression history and their depression state just prior to the study onset, some assumptions had to be made during the classification process. These assumptions were as follows:

1. **For the first prescription of an antidepressant within the study period,** the medication state (see Table 1 medication changes) was determined based on GP notes (general or prescription specific) with additional information supplied by PBS listings (i.e. recent prescription history) and the date of the first visit to the practice (i.e. whether they were a new patient at the time of the prescription). Two clinical desktop software programs were used by participating practices. One program (Medical Director (MD), developed by Health Communication Network) marked written prescriptions as either newly added, newly added and printed, or printed only. The second program (VIP, developed by Houston Medical Software) recorded prescriptions as printed only (i.e. no ‘added’ notation).

Where it was unclear and a decision had to be made between whether the prescription was to start (or restart) the antidepressant or to a maintain the dose, the following assumptions were used:

- 1. If the GP notes indicated the prescription had been newly added, or newly added and printed in MD, or it was recorded as printed in VIP, and there was
     - no recent prescription of the antidepressant supplied under the PBS => classify the prescription as start/restart.
     - a recent prescription of the antidepressant supplied under the PBS at
     - the same strength (mg) => maintain dose.
     - a different dose (mg) => check for further indications of change in dosage i.e. dose increase/decrease dose.
  2. If the prescription was listed as ‘printed’ in MD (i.e. not ‘added’), and there was
     - no recent prescription of the antidepressant supplied under the PBS => maintain dose (recognising that not all prescriptions are supplied under PBS).
     - a recent prescription of the antidepressant supplied under the PBS at
     - the same strength (mg) => maintain dose.
     - a different dose (mg) => check for further indications of change in dosage.

Note that ‘recent’ was considered to be within last 12 months.

1. **The state in which the patient began the study** was classified as follows:
   1. If there were no symptoms, no discussion of depression recorded, no subsequent information indicating a depressive state during this time, and there were
      - no current antidepressant prescriptions => recovery.
      - a current antidepressant prescription which was maintained, decreased or ceased => recovery.
      - a current antidepressant prescription which was maintained, decreased or ceased, and indications that the depressive state was being actively monitored (e.g. recording a normal score on a standardised depression assessment tool, such as the DASS 21) => remission
   2. If there were notes indicating initiation of an antidepressant within the previous two months, and a subsequent reduction in symptoms to mild levels => response
   3. If there were symptoms, or an increase in the antidepressant dose => depressive episode
2. **For patients experiencing depression with comorbid alcoholism**:
   1. Drinking was considered to indicate a depressive episode.
   2. Entering a detoxification centre for treatment of alcohol (or drug addiction) was not a recorded as a depression related hospitalisation as these providers were not included in SA Health hospitalisation data.

**Cycle correction adjustment**

A cycle correction was applied in order to account for the transition between states occurring between visits to the GP, rather than exactly on the visit date. The time between the visit at which the state changed and the visit prior, was taken as a transition period. It was assumed that the patient would visit the GP closer to the time of the more severe symptoms, therefore 25% of the days in the transition period were allocated to the more severe state. For example, if the patient transitioned from depressive episode to response, 25% of the days in the transition period were allocated to the depressive episode state. The remaining 75% of days were allocated to the response state.

Minimum durations for the response and remission states were enforced, consistent with the definitions of these states.

The classification process generated a series of GP visit dates with accompanying depression state classifications for each patient. From this, the number of days spent in each state could be calculated.

**Table 1 Categorisation of depressive state using routine data from general practice records**

|  | Depressive episode | Response | Remission | Recovery | Chronic depression | Hospital admission |
| --- | --- | --- | --- | --- | --- | --- |
| Medication changes |  |  |  |  |  |  |
| Start/restart AD^1^ | X |  |  |  |  |  |
| Increase dose | X | X |  |  |  |  |
| Switch AD^1^ | X | X |  |  |  |  |
| Combine AD^1^ | X | X |  |  |  |  |
| Maintain dose | X | X | X | X |  |  |
| Decrease dose^2^ |  |  |  | X |  |  |
| Taper off dose^2^ |  |  |  | X |  |  |
| Cease dose^2^ |  |  |  | X |  |  |
| Psychology/Psychiatry |  |  |  |  |  |  |
| Referral | X | X |  |  |  |  |
| Re-referral | X | X |  |  |  |  |
| Switch provider | X | X |  |  |  |  |
| Symptoms |  |  |  |  |  |  |
| Increased/new symptoms | X |  |  |  |  |  |
| Symptoms^3^ | X | X |  |  | X |  |
| Symptoms (>2yrs) |  |  |  |  | X |  |
| Improved symptoms^4^ | X | X |  |  | X |  |
| No symptoms recorded (<6mth) |  |  | X |  |  |  |
| No symptoms recorded (>6mth) |  |  |  | X |  |  |
| Other indicators |  |  |  |  |  |  |
| ED referral^5^ | X |  |  |  |  |  |
| Suicide attempt | X |  |  |  |  |  |
| Hospital admission |  |  |  |  |  | X |

^1^ AD: antidepressant. ^2^ providing change is not in response to side effects of medication. ^3^severity may range from minimal to significant.
^4^ improvement may range from minimal to significant. ^5^ ED: emergency department.
